# Association of mid‐trimester maternal angiogenic biomarkers with small‐for‐gestational‐age infants in an urban Zambian cohort: a nested case‐control study

**DOI:** 10.1002/ijgo.13860

**Published:** 2021-08-25

**Authors:** Chileshe M. Mabula‐Bwalya, Megan E. Smithmyer, Humphrey Mwape, Gabriel Chipili, Madelyn Conner, Bellington Vwalika, Kristina De Paris, Jeffrey S.A. Stringer, Joan T. Price

**Affiliations:** ^1^ University of North Carolina Global Projects Zambia Lusaka Zambia; ^2^ University of North Carolina at Chapel Hill Chapel Hill NC USA; ^3^ University of Zambia School of Medicine Lusaka Zambia

**Keywords:** birth weight, fetal growth, placental angiogenesis, small for gestational age, Zambia

## Abstract

**Objective:**

To investigate whether angiogenic biomarker concentrations differ between women who deliver small‐for‐gestational‐age (SGA) infants (<10th centile birth weight for gestational age) compared with controls, because identifying SGA risk early could improve outcomes.

**Methods:**

This case‐control study compared serum concentrations of angiogenic biomarkers before 24 weeks of pregnancy from 62 women who delivered SGA infants (cases) and 62 control women from an urban Zambian cohort. Odds of delivering an SGA infant were calculated using conditional logistic regression.

**Results:**

Placental growth factor (PlGF), soluble fms‐like tyrosine kinase (sFLT‐1) and soluble endoglin (sEng) in controls were 37.74 pg/mL (interquartile range [IQR] 23.12–63.15), 2525.18 pg/mL (IQR 1502.21–4265.54) and 2408.18 pg/mL (IQR 1854.87–3017.94), respectively. SGA cases had higher PlGF (40.50 pg/mL, IQR 22.81–67.94) and sFLT‐1 (2613.06 pg/mL, IQR 1720.58–3722.50), and lower sEng (2038.06 pg/mL, IQR 1445.25–3372.26). Participants with sEng concentration below and concomitant sFLT‐1 concentration above their respective thresholds (*n* = 40) had five‐fold higher odds of SGA (adjusted odds ratio 4.77, 95% confidence interval 1.61–14.1; *P* = 0.005).

**Conclusion:**

Biomarker concentrations were similar between cases and controls. Participants with concomitant low sEng and high sFLT‐1 had the highest odds of SGA, suggesting that a combination of biomarkers may better for predicting SGA than single biomarkers.

## Abstract 2

Prediction of SGA risk using the interaction of multiple angiogenic biomarkers may perform better than using a single angiogenic biomarker concentration before 24 weeks gestation.

## 1 INTRODUCTION

Low birth weight, which results from the overlapping conditions of prematurity and small for gestational age (SGA), contributes to over half of newborn deaths in sub‐Saharan Africa.^1^ Compared with neonates with a birth weight appropriate for gestational age, those born with a birth weight below the 10th centile for gestational age are at higher risk of neonatal mortality,^2^ poor neurocognitive outcomes,^3^ and chronic illness.^4^, ^5^ Although correct classification of SGA requires accurate gestational age estimation with ultrasound, largely unavailable to the world's most at‐risk women, globally, SGA affects 10% of neonates by definition, but is as high as 20% in low‐ and middle‐income countries.^6^ Resource scarcity further compounds the burden of SGA in settings with limited neonatal supportive technologies and long‐term care options.

Clinical detection of fetal growth restriction, a commonly used proxy for SGA outcome, is linked to decreased perinatal mortality.^7^ However, many infants at risk for growth restriction are missed by available methods of fetal surveillance, particularly in under‐resourced settings.^8^ The capacity to accurately identify women at risk of delivering SGA babies is therefore a matter of considerable clinical and public health importance worldwide.

Normal placental vascularization, angiogenesis, and growth rely on a tightly regulated signaling pathway involving the pro‐angiogenic factors vascular endothelial growth factor A (VEGF‐A) and placental growth factor (PlGF), along with binding proteins conventionally thought to have anti‐angiogenic activity, the soluble forms of endoglin (sEng) and fms‐like tyrosine kinase (sFLT‐1).^9^, ^10^ Dysregulation of these proteins is implicated in adverse birth outcomes, including hypertensive disorders, fetal growth restriction, and stillbirth.^11^, ^12^ Abnormal concentrations of these factors can be detected in maternal blood weeks before the clinical manifestation of obstetric disease,^12^, ^13^ and are potential targets for predictive tests of placentally‐derived pregnancy complications.

The current study aimed to assess whether mid‐trimester angiogenic biomarker concentrations were predictive of SGA in an urban Zambian cohort. We hypothesized that angiogenic biomarker concentrations would differ between women who delivered SGA neonates compared with those who delivered neonates that were either appropriate or large for gestational age.

## 2 MATERIALS AND METHODS

This was a case‐control study nested within the Zambian Preterm Birth Prevention Study (ZAPPS), an ongoing prospective observational cohort of pregnant women in Lusaka, Zambia. Cohort characteristics and key outcomes have been described in detail elsewhere.^14^, ^15^ Briefly, 1450 pregnant women were enrolled at a median gestational age of 16 weeks (interquartile range [IQR] 13–18 weeks) between August 2015 and September 2017. Venous whole blood specimens were collected from participants at study visits. Blood was centrifuged at 3000 g for 5 minutes within 2 hours of collection, and the resultant serum was divided into aliquots and stored at –80℃. Participants were followed through delivery, and the neonate's vital status, sex, birth weight, gestational age, and mode of delivery were recorded.

**FIGURE 1:**
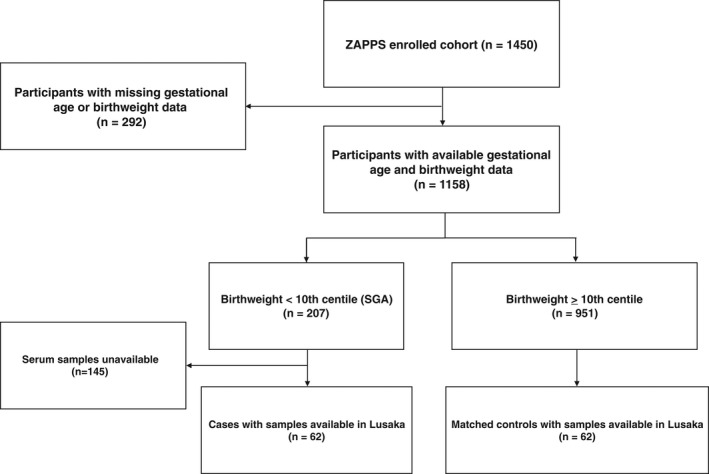
Study sample selection flow diagram

The current study included women pregnant with a single fetus, with a serum sample collected before 24 weeks of pregnancy, and who had data recorded on both gestational age and birth weight (Figure 1). Cases were defined as women who delivered an infant with a birth weight below the 10th centile for gestational age as calculated by INTERGROWTH‐21st standards,^16^ whereas controls were defined as those who delivered neonates with a birth weight at or above the 10th centile. Gestational age was calculated using ultrasound biometry at enrollment. Controls were matched to cases in a 1:1 ratio based on maternal height (±10 cm), HIV serostatus, and gestational age at serum sample collection (±7 days). Sample size for this exploratory analysis was dictated by the availability of candidate samples in the Lusaka biorepository.

The use of clinical data and biological specimens for research purposes was approved by the University of Zambia Biomedical Research Ethics Committee, the University of North Carolina at Chapel Hill Institutional Review Board, and the Zambian Ministry of Health. Demographic data and biological samples were de‐identified, and all women provided written informed consent before enrollment for participation in the cohort and associated laboratory analyses.

Angiogenic biomarker concentrations in thawed serum samples were quantified using multiplex bead array assays (Magnetic Luminex Assay Human Premixed Multi‐Analyte Kit; R&D Biosystems) according to the manufacturer's instructions. Serum VEGF‐A was assessed on a separate well‐plate from PlGF, sFLT‐1, and sEng, due to the binding between VEGF‐A and sFLT‐1, a VEGF receptor. A Luminex MAGPIX Analyzer was used to determine biomarker concentration, which was captured using xponent 4.2 software (Luminex Corporation). A minimum concentration of 50 microspheres was used as the threshold for the calculation of mean fluorescence intensity. Laboratory staff performing assays were blinded to clinical outcome.

Frequencies and distributions of baseline covariates between cases and controls were compared using χ^2^ test for categorical variables and either independent sample *t* test or Mann‐Whitney *U* test for continuous comparisons, depending on normality of distribution. Mann‐Whitney *U* tests were used to compare concentrations of VEGF‐A, PlGF, sFLT‐1, and sEng between the SGA group and controls. Ratios of biomarker concentration for PlGF/sEng, PlGF/sFLT‐1, and sEng/sFLT‐1 were also calculated and compared between cases and controls. Values of *P* < 0.05 were considered statistically significant.

We transformed continuous biomarker concentrations into quartiles and evaluated the odds of SGA by quartile for each biomarker. Threshold analysis was used to identify whether a single cut‐point in each biomarker concentration predicted SGA, and to explore the effect of combining threshold concentrations that individually predicted SGA in single threshold analysis. To evaluate non‐linear relationships between individual biomarker concentrations and the SGA outcome, we applied restricted cubic splines, using Akaike's information criterion to choose an appropriate number of knots.^17^

Conditional logistic regression was used to estimate the odds of delivering an SGA neonate. Covariates were selected on the basis of clinical relevance, significant association with SGA at an α level <0.2 in crude analysis, or a change in the regression coefficient in any model by more than 10%. Data management and analysis were performed using STATA (version 16.1; StataCorp LP).

## 3 RESULTS

A total of 124 participants were included in this analysis: 62 cases who delivered SGA infants and 62 controls (Table 1). Median gestational age at sample collection was 18.3 weeks (IQR 17.4–19.6 weeks). More controls (*n* = 51/62, 82%) were married or cohabiting compared with cases (*n* = 46/62, 74%; *P* = 0.2). Additionally, a modestly higher proportion of mothers who delivered SGA infants were nulliparous (*n* = 29/62, 47%) compared with controls (*n* = 23/62, 37%; *P* = 0.3). No significant between‐group differences were noted in the prevalence of hypertension or anemia at enrollment, nor for vaginal bleeding in any trimester. Overall, 10 women (8%) were HIV seropositive at enrollment, with even distribution between cases and controls.

**TABLE 1:** Baseline characteristics of cases (SGA <10th centile) and controls (*N* = 124)^a^

Characteristic	All (*N* = 124)	Not SGA (*n* = 62)	SGA (*n* = 62)	*P* b
Age, years	25 (22–30)	25 (23–30)	25 (21–30)	0.3
<20	15 (12.1)	5 (8.2)	10 (16.1)	
20–34	96 (77.4)	49 (80.3)	47 (75.8)	
≥35	12 (9.7)	7 (11.5)	5 (8.1)	
Missing	1	1	0	
Married or cohabiting	97 (78.2)	51 (82.3)	46 (74.2)	0.2
Missing	1	1	0	
Parity	1 (0–2)	1 (0–2)	1 (0–2)	0.5
Nulliparous	52 (41.9)	23 (37.1)	29 (46.8)	
EGA, weeks	18.3 (17.4–19.6)	18.3 (17.4–19.6)	18.4 (17.3–19.4)	>0.9
Maternal height, cm	159 (154–163)	160 (155–165)	158 (153–163)	0.2
Maternal weight, kg	61 (53–66)	63 (54–74)	58 (53–66)	0.02
Missing	2	1	1	
BMI	18 (17–20)	18 (17–20)	18 (17–19)	0.08
<18.5	1 (0.8)	0 (0)	1 (1.6)	
18.5–30.0	104 (86.7)	52 (86.7)	52 (86.7)	
>30	15 (12.5)	8 (13.3)	7 (11.7)	
Missing	4	2	2	
MUAC, cm	28 (26–30)	28 (27–30)	28 (26–29)	0.05
Hypertensive	3 (2.5)	1 (1.6)	2 (3.3)	
Missing	2	1	1	
HIV seropositive	10 (8.1)	5 (8.1)	5 (8.1)	>0.9
Syphilis seropositive	0 (0)	0 (0)	0 (0)	–
Hemoglobin, mg/dL	12 (11–13)	12 (11–13)	12 (11–13)	>0.9
<10.5	15 (18.1)	8 (18.6)	7 (17.5)	
Missing	30	19	11	
Bacteriuria^c^	2 (1.7)	1 (1.7)	1 (1.7)	>0.9
Missing	7	4	3	

Abbreviations: BMI, body mass index (calculated as weight in kilograms divided by the square of height in meters); EGA, estimated gestational age; IQR, interquartile range; MUAC, mid‐upper arm circumference; SD, standard deviation; SGA, small for gestational age <10th centile, TM, trimester.

^a^ Values are presented as number (percentage) or as median (interquartile range).

^b^ *P* values calculated by Mann‐Whitney *U* test for continuous and by χ^2^ for categorical comparisons.

^c^ Defined as ≥1+ leukocyte esterase or +nitrites on urine dip.

Although participants were matched on height, there were notable differences in maternal weight at enrollment between cases (median 58 kg, IQR 53–66 kg) and controls (median 63 kg, IQR 54–74 kg; *P* = 0.02). Similarly, mid‐upper arm circumference was marginally smaller among women who delivered SGA infants (median 28 cm, IQR 26–29 cm) compared with controls (median 28 cm, IQR 27–30 cm; *P* = 0.05).

Among SGA cases, median birth weight was 2600 g (IQR 2370–2795 g) and centile was 4.4 (IQR 2.5–6.7), whereas among controls, median birth weight was 3200 g (IQR 3000–3500 g) and centile was 40.9 (IQR 23.8–67.5). Median gestational age at delivery was similar between cases and controls.

**FIGURE 2:**
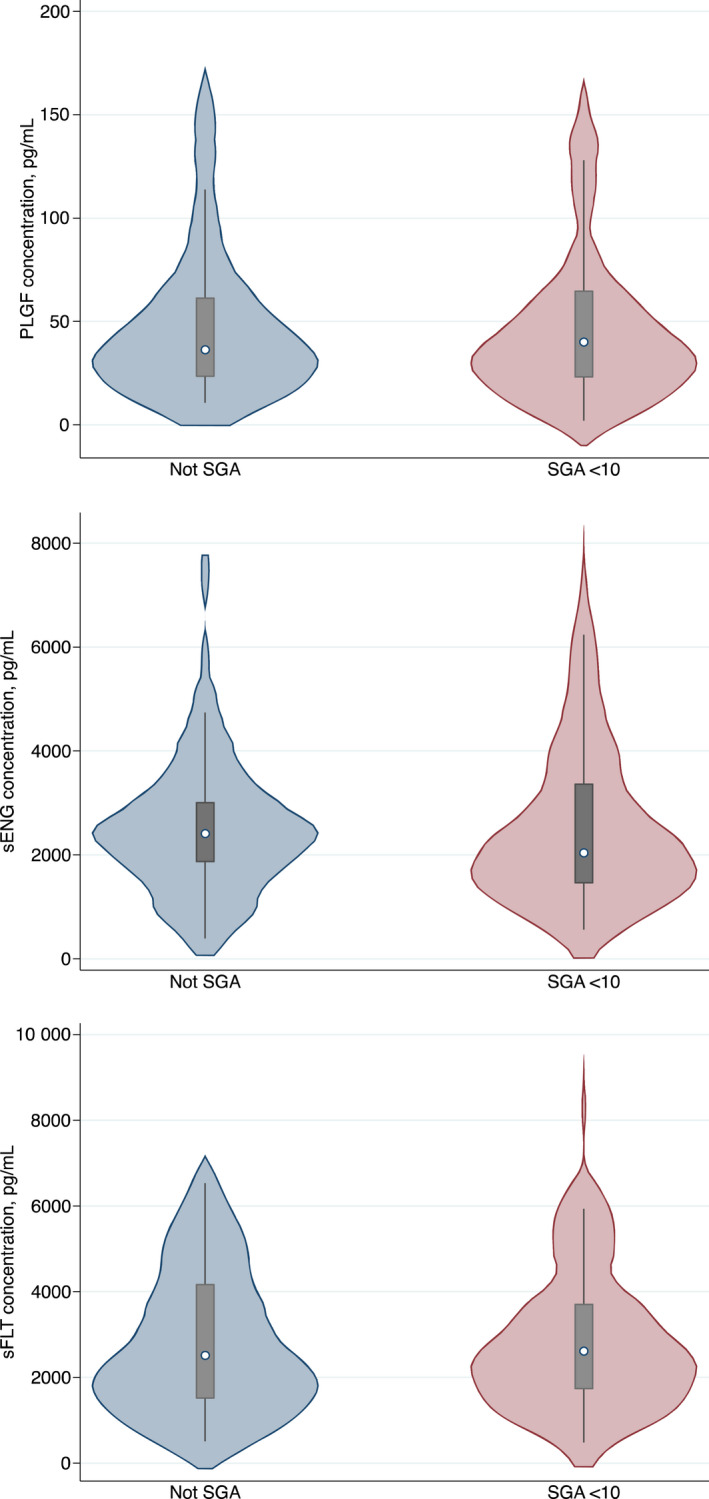
Concentrations of maternal serum angiogenic biomarkers (pg/mL) between infants born small for gestational age (SGA) and controls, *N* = 124 [Colour figure can be viewed at wileyonlinelibrary.com]

The median values of PlGF, sFLT‐1, and sEng in controls were 37.74 pg/mL (IQR 23.12–63.15 pg/mL), 2525.18 pg/mL (IQR 1502.21–4265.54 pg.mL) and 2408.18 pg/mL (IQR 1854.87–3017.94 pg/mL), respectively. Women who delivered an SGA infant had numerically higher PlGF (40.50 pg/mL, IQR 22.81–67.94 pg/mL) and sFLT‐1 (2613.06 pg/mL, IQR 1720.58–3722.50 pg/mL), and lower sEng (2038.06 pg/mL, IQR 1445.25–3372.26 pg/mL); however, between‐group differences in biomarker concentrations did not reach statistical significance (Table 2; Figure 2). Ratios of PlGF/sEng, PlGF/sFLT‐1, and sEng/sFLT‐1 were also similar between cases and controls (Table 3). VEGF‐A levels were below minimum detectable levels and were excluded from analysis.

**TABLE 2:** Concentrations of angiogenic biomarkers between cases (SGA) and controls (*N* = 124)

	Not SGA (*n* = 62)			SGA (*n* = 62)			
	Median	p25	p75	Median	p25	p75	*P* a
Biomarker concentration, pg/mL							
PlGF	37.74	23.12	63.15	40.50	22.81	67.94	0.9
sFLT	2525.18	1502.21	4265.54	2613.06	1720.58	3722.50	0.9
sEng	2408.18	1854.87	3017.94	2038.06	1445.25	3372.26	0.6
Ratio of biomarker concentrations							
PlGF/sEng	0.02	0.01	0.03	0.02	0.01	0.04	0.9
PlGF/sFLT	0.02	0.01	0.03	0.02	0.01	0.03	0.9
sEng/sFLT	0.95	0.57	1.32	0.82	0.55	1.36	0.7

Abbreviations: PlGF, placental growth factor; sFLT, soluble fms‐like tyrosine kinase; sEng, soluble endoglin; SGA, small for gestational age <10th centile.

^a^ All *P* values calculated by Mann‐Whitney *U* test between cases and controls

**TABLE 3:** Odds of SGA by quartiles and thresholds of individual angiogenic biomarker concentrations (*N* = 124)

	*N*	Median (IQR)	No. of events	%	Not SGA			SGA		
					OR	95% CI	*P*	aOR^a^	95% CI	*P*
PlGF, pg/mL										
Quartile 1 (ref)	31	16.1 (13.2–19.3)	16	52	1	–	–	1	–	–
Quartile 2	31	29.6 (26.2–34.9)	14	45	0.77	0.28–2.10	0.6	0.38	0.10–1.39	0.1
Quartile 3	31	48.8 (45.3–55.9)	16	52	1	–	–	1.34	0.31–5.91	0.7
Quartile 4	31	118.0 (82.8–145.8)	16	52	1	–	–	0.73	0.16–3.31	0.7
Threshold <115 pg/mL	108	34.3 (21.4–50.3)	52	48	0.50	0.15–1.68	0.3	0.24	0.06–1.00	0.05
sFLT−1, pg/mL										
Quartile 1 (ref)	31	1234.4 (861.5–1365.4)	12	39	1	–	–	1	–	–
Quartile 2	32	1956.1 (1762.2–2125.4)	19	59	2.18	0.78–6.06	0.1	2.76	0.64–11.9	0.2
Quartile 3	30	3124.4 (2870.3–3338.0)	18	60	2.29	0.75–7.02	0.1	1.68	0.41–6.93	0.5
Quartile 4	31	5464.0 (4611.7–5922.8)	13	42	1.03	0.36–2.95	>0.9	0.79	0.21–2.91	0.7
Threshold <1360 pg/mL	23	1076.2 (818.2–1290.0)	8	35	0.46	0.17–1.22	0.1	0.33	0.10–1.15	0.08
sEng, pg/mL										
Quartile 1 (ref)	31	1202.9 (695.7–1382.4)	17	55	1	–	–	1	–	–
Quartile 2	31	1955.4 (1771.2–2055.4)	18	58	1.03	0.38–2.83	>0.9	0.76	0.23–2.48	0.7
Quartile 3	32	2576.7 (2408.2–2862.6)	9	28	0.28	0.10–0.80	0.02	0.17	0.07–0.81	0.02
Quartile 4	30	4202.6 (3668.3–5517.0)	18	60	1.47	0.56–3.84	0.4	1.62	0.43–6.15	0.5
Threshold <2075 pg/mL	56	1445.3 (1128.4–1868.3)	34	61	1.92	0.98–3.78	0.06	2.34	1.11–4.97	0.03

Abbreviations: aOR, adjusted odds ratio; CI, confidence interval; IQR, interquartile range; OR, odds ratio; PlGF, placental growth factor; sEng, soluble endoglin; sFLT, soluble fms‐like tyrosine kinase; SGA, small for gestational age <10th centile.

^a^ Conditional logistic models adjusted for parity, marital status, maternal weight at enrollment, and hypertension at enrolment.

Conditional logistic regression revealed that sEng concentrations in the third quartile (range 2219.43–3254.04 pg/mL) were associated with lower odds of delivery of an SGA infant compared with the lowest quartile (odds ratio [OR] 0.28, 95% confidence interval [CI] 0.10–0.80, *P* = 0.02) (Table 3). This protective effect remained when the model was adjusted for parity, marital status, maternal weight, hypertension, and infant sex (aOR 0.17, 95% CI 0.07–0.81, *P* = 0.02). In threshold analysis, sEng concentrations below 2075 pg/mL were associated with two‐fold higher odds of SGA (adjusted OR [aOR] 2.34, 95% CI 1.11–4.97; *P* = 0.03). We found a borderline protective effect of low PlGF (aOR 0.24, 95% CI 0.06–1.00; *P* = 0.05) and low sFLT‐1 (aOR 0.33, 95% CI 0.10–1.15; *P* = 0.08) on SGA.

**FIGURE 3:**
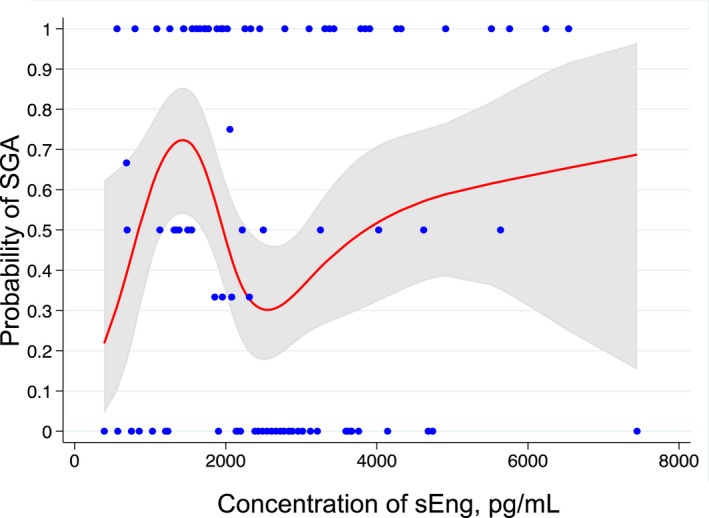
Restricted cubic spline of soluble endoglin (sEng) concentration (pg/mL) by probability of small for gestational age <10th centile (SGA), *N* = 124 [Colour figure can be viewed at wileyonlinelibrary.com]

Analysis of restricted cubic splines revealed a non‐linear relationship between sEng concentration and SGA (*P* = 0.04) (Figure 3). The odds of SGA increased with increasing sEng concentrations up to approximately 1500 pg/mL and decreased again between 1500 and 3000 pg/mL. When sEng concentrations rose above 3000 pg/mL, SGA odds gradually increased to near equivalence of those experienced at 1500 pg/mL. Cubic spline analyses of the odds of SGA by other biomarker levels were not informative.

Participants with a mid‐trimester sEng concentration below and concomitant sFLT‐1 concentration above their respective thresholds (*n/N* = 28/40) had nearly five‐fold higher odds of delivering an SGA neonate (aOR 4.77, 95% CI 1.61–14.1; *P* = 0.005). Similarly, participants with low PlGF, low sEng, and high sFLT‐1 (*n/N* = 24/36) on threshold analysis had higher odds of SGA compared with the rest of the cohort (aOR 3.16, 95% CI 1.13–8.85; *P* = 0.03) (Table 4).

**TABLE 4:** Odds of small for gestational age (SGA) by combined thresholds of angiogenic biomarker concentrations

	SGA (*n*/*N*)	%	OR	95% CI	*P* value	aOR^a^	95% CI	*P* value
Low sEng and high sFLT	28/40	70	4.33	1.22–15.36	0.02	4.77	1.61–14.1	0.005
Low PlGF, low sEng, high sFLT	24/36	67	2.50	1.09–5.71	0.03	3.16	1.13–8.85	0.03

Abbreviations: aOR, adjusted odds ratio; CI, confidence interval; OR, odds ratio; PlGF, placental growth factor; sEng, soluble endoglin; sFLT, soluble fms‐like tyrosine kinase; SGA, small for gestational age <10th centile.

^a^ Conditional logistic model adjusted for parity, marital status, maternal weight at enrollment, hypertension at enrollment, and infant sex.

## 4 DISCUSSION

In this nested case‐control study of pregnant Zambian women, we found similar concentrations of PlGF, sFLT‐1, and sEng between women who delivered an SGA neonate compared with those who did not. Maternal serum sEng concentration in the third quartile was associated with lower odds of delivering an SGA infant; this non‐linear association between sEng and SGA was confirmed by spline analysis. The highest odds of SGA were found among participants with concomitant low sEng, and high sFLT‐1, with or without low PlGF. This suggests that a combination of more than one angiogenic biomarker with consideration of thresholds may better predict growth restriction than any single biomarker or fixed value, which is consistent with findings from previous studies.^18^

Our finding of a non‐linear relationship between sEng concentration and the odds of an SGA delivery supports the existence of an optimal range for sEng concentration. Transmembrane endoglin and possibly sEng may produce an anti‐angiogenic effect by limiting the availability of members of the transforming growth factor‐β (TGF‐β) family. Two different pathways with opposite effects may be stimulated by TGF‐β, depending on concentration and environment. The ALK‐1 pathway induces angiogenesis through the proliferation and migration of endothelial cells, whereas the ALK‐5 pathway has the reverse effect.^19^ Outside the optimal range, sEng concentration may cause TGF‐β to shift towards either a pro‐angiogenic pathway or an anti‐angiogenic pathway in a dose‐dependent manner. Given that these biomarkers have complicated pathways that may act in opposing manners based on the physiological circumstance, further studies are warranted to fully elucidate their mechanisms of action and downstream effects.^20^

The similarities in the overall concentrations of angiogenic biomarkers between cases and controls may suggest that other mechanisms of poor fetal growth that are prevalent in low‐ and middle‐income countries may contribute to birthweight below the tenth centile in our cohort. SGA includes both constitutionally small but healthy neonates and those whose growth was restricted in utero due to placental dysfunction, maternal conditions, or fetal genetic abnormalities. The International Fetal and Newborn Growth Consortium (INTERGROWTH‐21^st^) study demonstrated similar fetal growth in eight geographically defined urban populations, when controlled for nutrition, antenatal care, and environmental growth constraints, suggesting that at least some SGA is modifiable.^16^, ^21^ Inflammation from malnutrition, infection, or helminth infestation has been implicated in the pathogenesis of several adverse obstetric outcomes,^22^, ^23^ but might not generate the placental angiogenic profile traditionally associated with SGA. An investigation of circulating sEng levels in pregnant women from Malawi and Cameroun reported higher levels of sEng in primigravid women—the demographic that trended towards higher incidence of SGA in this sample—and showed higher sEng concentrations with both infection and fetal growth restriction.^24^ Although this report contrasted with our results, it potentially supports the existence of alternative pathways. Maternal constitutional smallness and malnutrition are associated with SGA^34^ and may be predictors of fetal growth independent of placental angiogenic biomarkers; this is supported by our observation that women who delivered SGA infants trended towards lower maternal weight and mid‐upper arm circumference compared with controls.

Previous studies with similar gestational ages have reported conflicting findings for the association between angiogenic biomarker concentration and SGA. A systematic review of 26 studies assessing the roles of sFLT‐1 and PlGF in the prediction of SGA found minimal differences in the concentrations of these biomarkers in SGA and controls. The largest differences for sFLT‐1 and PlGF were reported after 26 weeks gestational age, well after our study's median gestational age and cut‐off point.^25^ Our findings may reflect the subtlety of changes in the early second trimester window we selected, and the placenta's ability to compensate for derangements during this stage of pregnancy. Differences in levels of these biomarkers may only become more pronounced at later time points in pregnancy.

A major strength of this study was its primary focus on SGA, conducted in a well‐characterized cohort of African women. Much of the data on maternal angiogenic biomarkers and SGA has been derived from European and North American cohorts; data from low‐ and middle‐income country populations are sparse. Two recent systematic reviews of angiogenic and inflammatory biomarkers to predict SGA included a single mid‐trimester study^26^ from a low‐ and middle‐income country out of a combined 28  trials.^25^, ^27^ The non‐uniform risk of placentally‐driven disease across race, ethnicity, and geography^28^, ^29^, ^30^, ^31^ underscores the importance of further study in diverse populations. An added strength of our study is the use of ultrasound biometry for gestational age estimation, a rarity in antenatal cohorts in the region that is critical for accurate classification of SGA.

Our study acknowledges limitations. Our use of the 10th centile for SGA, due to the fact that the proportion of neonates in our cohort with SGA below the third centile was too small for a matched case‐control analysis, may have limited the proportion of cases resulting from angiogenic dysregulation compared with constitutional smallness and pathological SGA driven by non‐placental factors. Some SGA biomarker studies that reported a significant between‐group difference employed birth weights below the 5th^18^, ^32^ or 2.5th centile,^33^ which likely concentrated any discernible effect. Additionally, although our sample size for this exploratory analysis was within the range of many studies of biomarker prediction of SGA,^25^ our wide confidence intervals indicate that future studies with larger sample sizes are needed.

Our results suggest that single angiogenic biomarker concentrations before 24 weeks of gestation may be of limited utility, but risk stratification using interactions of multiple biomarkers may perform better. Ethnogeographic variations in the relationship between angiogenic biomarkers and fetal growth restriction merit further study. Discovery of assays that can accurately identify those fetuses at highest risk for SGA early in gestation could improve management and reduce adverse neonatal outcomes.

## CONFLICTS OF INTEREST

The authors have no conflicts of interest.

## AUTHOR CONTRIBUTIONS

CMB, BV, JSAS, and JTP conceptualized and obtained funding for this study. CMB, MES, HM, and GC performed laboratory analyses and data management. CMB, MES, BV, MC, MPK, BV, KDP, JSAS, and JTP contributed to the design and interpretation of the analysis. CMB and JTP conducted the statistical analysis and co‐wrote the manuscript. All co‐authors reviewed the manuscript, provided critique of the intellectual content, and approved the final version before submission.
